# Raw-Data Driven Functional Data Analysis with Multi-Adaptive Functional Neural Networks for Ergonomic Risk Classification Using Facial and Bio-Signal Time-Series Data

**DOI:** 10.3390/s25154566

**Published:** 2025-07-23

**Authors:** Suyeon Kim, Afrooz Shakeri, Seyed Shayan Darabi, Eunsik Kim, Kyongwon Kim

**Affiliations:** 1 Department of Statistics, Ewha Womans University, Seoul 03760, Republic of Korea; suy@ewhain.net; 2 Department of Mechanical, Automotive, and Materials Engineering, University of Windsor, Windsor, ON N9B 3P4, Canada; shakeri1@uwindsor.ca (A.S.); darabi@uwindsor.ca (S.S.D.); eskim@uwindsor.ca (E.K.); 3 Department of Applied Statistics, Department of Statistics and Data Science, Yonsei University, Seoul 03722, Republic of Korea

**Keywords:** functional data analysis, neural network, classification

## Abstract

Ergonomic risk classification during manual lifting tasks is crucial for the prevention of workplace injuries. This study addresses the challenge of classifying lifting task risk levels (low, medium, and high risk, labeled as 0, 1, and 2) using multi-modal time-series data comprising raw facial landmarks and bio-signals (electrocardiography [ECG] and electrodermal activity [EDA]). Classifying such data presents inherent challenges due to multi-source information, temporal dynamics, and class imbalance. To overcome these challenges, this paper proposes a Multi-Adaptive Functional Neural Network (Multi-AdaFNN), a novel method that integrates functional data analysis with deep learning techniques. The proposed model introduces a novel adaptive basis layer composed of micro-networks tailored to each individual time-series feature, enabling end-to-end learning of discriminative temporal patterns directly from raw data. The Multi-AdaFNN approach was evaluated across five distinct dataset configurations: (1) facial landmarks only, (2) bio-signals only, (3) full fusion of all available features, (4) a reduced-dimensionality set of 12 selected facial landmark trajectories, and (5) the same reduced set combined with bio-signals. Performance was rigorously assessed using 100 independent stratified splits (70% training and 30% testing) and optimized via a weighted cross-entropy loss function to manage class imbalance effectively. The results demonstrated that the integrated approach, fusing facial landmarks and bio-signals, achieved the highest classification accuracy and robustness. Furthermore, the adaptive basis functions revealed specific phases within lifting tasks critical for risk prediction. These findings underscore the efficacy and transparency of the Multi-AdaFNN framework for multi-modal ergonomic risk assessment, highlighting its potential for real-time monitoring and proactive injury prevention in industrial environments.

## 1. Introduction

Work-related musculoskeletal disorders frequently arise from improper or hazardous lifting techniques. Accurate classification of ergonomic risk levels associated with lifting tasks is essential for injury prevention in industrial and clinical contexts. Traditional methods of ergonomic risk assessment, such as observational checklists or standardized formulas (e.g., the National Institute for Occupational Safety and Health (NIOSH) lifting index), are often subjective and labor-intensive. Conversely, automated risk classification leveraging sensor data offers a promising approach for objective and continuous ergonomic monitoring. Previous studies employing wearable sensors, such as electromyography (EMG) and inertial measurement units, combined with deep learning techniques have demonstrated promising outcomes primarily for binary risk classification (safe versus unsafe lifting). However, distinguishing between multiple risk levels (low, medium, high) remains challenging due to subtle signal variations and inherent class imbalance, given that serious injuries occur relatively infrequently.

Multi-modal data fusion can potentially enhance ergonomic risk assessment by capturing complementary indicators of strain. For instance, facial expressions and muscle activations provide visible signs of effort or discomfort, while bio-signals, including heart rate, respiration, and EMG, quantify physiological stress responses. Although integrating these modalities may improve classification accuracy, it introduces several challenges related to the analysis of noisy and temporally variable time-series data. Standard feature concatenation techniques may not adequately exploit temporal dependencies. Furthermore, conventional sequential models, such as convolutional neural networks (CNNs) and long short-term memory (LSTM) networks, typically require large datasets and provide limited interpretability, making it challenging to discern specific signal components associated with risk.

Functional data analysis (FDA) offers a systematic approach to handle time-series data by modeling them as continuous functions. FDA methods typically employ basis function expansions such as splines and Fourier series to represent temporal data, facilitating the classification of functional observations. Methods such as functional discriminant analysis and logistic regression leverage functional principal components or basis coefficients to achieve classification, exploiting signal smoothness to highlight significant temporal regions. However, traditional FDA methods often rely on predefined basis functions (e.g., splines or wavelets), potentially restricting their ability to capture task-specific relevant features. Recent advancements have integrated FDA with deep learning frameworks to overcome these limitations. Adaptive Functional Neural Networks (AdaFNNs) represent an innovative approach wherein custom basis functions are learned directly from data through adaptive basis layers composed of micro-networks, significantly enhancing classification accuracy compared to fixed-basis FDA approaches.

This paper proposes the Multi-Adaptive Functional Neural Network (Multi-AdaFNN), an extension of AdaFNN designed specifically for multi-modal ergonomic risk classification of lifting tasks using synchronized raw facial landmarks and bio-signal (ECG and EDA) time-series data. The proposed method offers the following contributions: (1) the development of Multi-AdaFNN, which assigns dedicated adaptive micro-networks to each individual time-series feature, effectively concatenating the learned basis coefficients for accurate risk classification; (2) the introduction of a novel laboratory dataset comprising manual-lifting trials categorized into low, medium, or high risk, with five distinct input configurations—facial landmarks only, bio-signals only, full fusion, selected facial landmarks, and selected facial landmarks combined with bio-signals—to investigate sensor fusion and feature selection effects; (3) rigorous evaluation of model performance using 100 independent stratified splits (70% training and 30% testing), demonstrating consistently superior outcomes with fused multi-modal inputs compared to single-modality configurations; and (4) a discussion of practical implications, current limitations, and future research directions, including comparative benchmarking against conventional classifiers and deployment considerations for real-time ergonomic risk assessment.

Section 2 outlines dataset specifics, including participants, data collection protocols, signal preprocessing techniques, and experimental input configurations. Section 3 formulates the classification task, details the proposed Multi-AdaFNN architecture, introduces the generalized sparse additive model (GSAM), and describes the training procedures, hyper-parameter tuning, and evaluation protocol. Section 4 presents results across evaluated dataset configurations using metrics such as accuracy, weighted F1-score, and area under the receiver operating characteristic (ROC) curve (AUC) and includes a comparative analysis against an LSTM baseline. Section 5 discusses the implications, strengths, limitations, and avenues for future work. Finally, Section 6 summarizes key findings and emphasizes the potential application of Multi-AdaFNN for real-time ergonomic risk monitoring in workplace environments.

## 2. Dataset

### 2.1. Participants

In a controlled laboratory experiment designed to simulate lifting tasks, twenty participants, comprising an equal number of males and females, were recruited. Female participants had a mean age of 28.2 years (SD = 4.29), while males had a mean age of 26.8 years (SD = 4.83). On average, female participants measured 165.4 cm (SD = 4.58) in height and weighed 59.7 kg (SD = 8.76), whereas male participants had an average height of 178.4 cm (SD = 6.65) and an average weight of 79.8 kg (SD = 22.60). All participants provided informed consent prior to participation, in accordance with the ethical guidelines of the University of Windsor and approval from the Research Ethics Board (REB# 23-072).

### 2.2. Equipment and Data Collection

In this study, a custom-designed lifting simulator was employed to enable participants to lift a handle connected to adjustable weights from predetermined heights and distances to simulate various lifting tasks. The simulator comprised two height-adjustable stands—one shorter and one taller—to establish different lifting elevations. Clearly marked spots on the platform guided participants in maintaining consistent foot placements according to the experimental conditions. Participants positioned themselves on these spots and lifted a handle serving as the load. Resistance was regulated by adjusting weights attached to the opposite end of a cable, which was routed through reels, facilitating precise control over lifting difficulty while maintaining consistent experimental conditions.

Participants’ facial expressions during the lifting tasks were recorded using a head-mounted camera setup, featuring a GoPro camera attached to a helmet via an adjustable arm. This arrangement ensured optimal camera positioning without obstructing facial visibility and facilitated stable video recordings at a frame rate of 30 frames per second.

Additionally, physiological bio-signals were recorded utilizing the biosignalsplux Hybrid-8 toolkit at a sampling frequency of 300 Hz. A total of five electrodes were used—three for ECG and two for EDA. ECG electrodes were positioned below the left and right collarbones and on the lower left ribcage, while the back of the neck was selected for EDA recording due to its high density of sweat glands and minimal interference with lifting movements, as recommended by Hossain et al. [1].

### 2.3. Experiment Design

Various lifting tasks were systematically simulated by manipulating two critical factors: horizontal distance (HD) and vertical distance (VD). These parameters were specifically chosen due to their substantial impact on the recommended weight limit (RWL) and their association with lower back pain (LBP) risk. For instance, the RWL is maximized when the load is placed approximately 25 cm away from the body, whereas doubling this distance significantly reduces the RWL by half. Similarly, lifting loads from the ground level can double spinal load compared to lifting from an optimal height, thereby increasing the risk of developing LBP [2]. Accordingly, four distinct lifting scenarios were established by varying HD and VD parameters, while all other factors of the revised NIOSH lifting equation (RNLE) remained constant. In particular, HD values were set at 25 cm (optimal) and 50 cm (suboptimal), whereas VD was designated as 75 cm (optimal knuckle height) and 10 cm (suboptimal ground level). Table 1 summarizes these lifting conditions, defined by their unique combinations of HD and VD.

The Vertical Travel Distance (VTD) was standardized at 65 cm to simulate lifts from ground to knuckle height and from knuckle to shoulder height. No trunk rotation or twisting movements were included, and thus, the Asymmetry Angle (AA) was set to zero degrees. Participants used a specially designed S-shaped handle providing a secure grip, resulting in a coupling quality rated as optimal. The lifting frequency was uniformly maintained at four lifts per minute across all conditions.

To determine the RWL, multipliers from RNLE were computed for each lifting scenario. Since RWL was constant within each condition, the actual load weight (LW) was adjusted to achieve varying lifting index (LI) levels, facilitating the simulation of safe (LI < 1) and risky (LI > 1) tasks. Specifically, conditions 1 and 3 included one safe and one risky task each, while conditions 2 and 4 comprised one safe task and two risky tasks—one with LI between 1 and 2 and another with LI between 2 and 3. Tasks requiring loads heavier than 23 kg (the maximum allowable load under ideal RNLE conditions) were excluded from conditions 1 and 3 [3].

**Figure 1:**
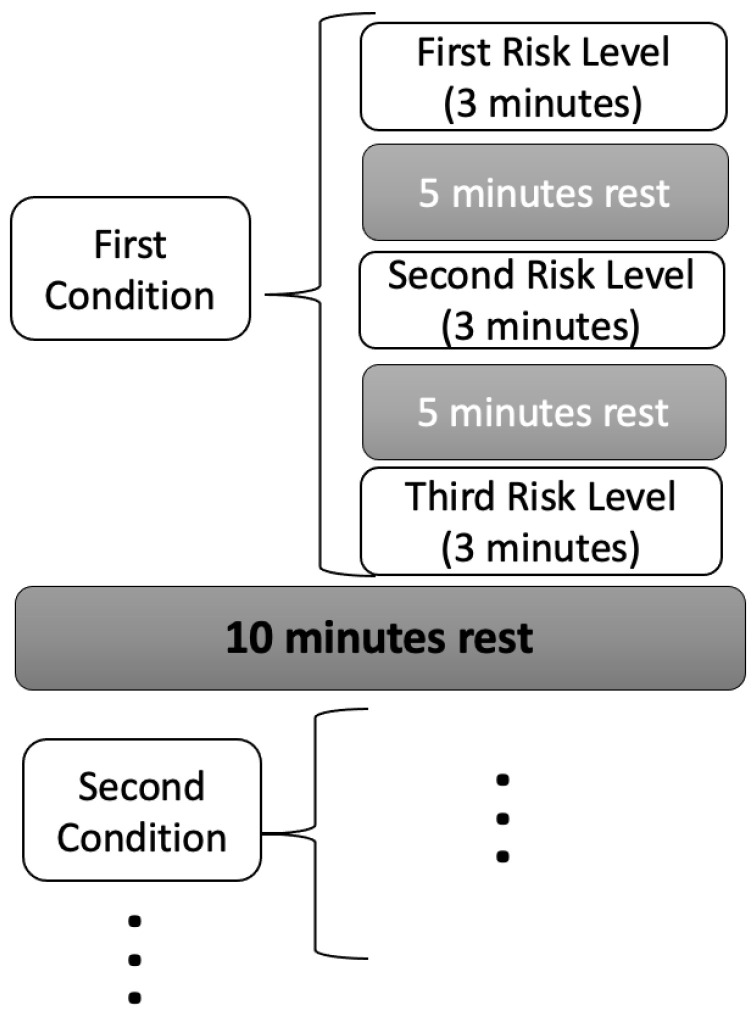
Sequence and structure of the lifting tasks: ten three-minute sessions targeting twelve lifts each (with early termination allowed to prevent excessive fatigue), interspersed with at least five minutes of rest within conditions and at least ten minutes of rest between conditions.

Each participant completed a total of ten lifting tasks, with each task lasting three minutes, during which participants aimed to perform twelve lifts. To prevent excessive fatigue, participants were permitted to stop prematurely if necessary. Rest periods of at least five minutes were provided between tasks within the same condition, while intervals of at least ten minutes separated tasks across different conditions, with additional rest allowed upon request. Comprehensive details regarding experimental conditions, tasks, and associated risks are summarized in Table 2. Additionally, Figure 1 illustrates the sequence and structure of the lifting tasks, offering a clear overview of the experimental task patterns. Load weights were randomly allocated within the appropriate LI range for each participant and task order was randomized.

### 2.4. Facial Dataset

To quantify temporal changes in facial expressions, variations in facial landmark positions were systematically analyzed utilizing computer vision techniques. Specifically, the MediaPipe framework was employed due to its demonstrated accuracy in facial landmark detection [4]. MediaPipe detects a total of 468 facial landmarks, each characterized by corresponding *x* and *y* coordinates across the facial region.

To capture dynamic changes in facial expressions, the Euclidean distance between coordinates of each facial landmark from consecutive frames was computed. For example, the distance between the coordinates $\left(x_{1},y_{1}\right)$ of landmark 1 in frame 1 and coordinates $\left(x_{2},y_{2}\right)$ in frame 2 is calculated using the following formula:

(1) $$\mathrm{distance}=\sqrt{{\left(x_{2}-x_{1}\right)}^{2}+{\left(y_{2}-y_{1}\right)}^{2}}.$$

To prepare a uniform dataset, the length of the longest video (5282 frames) was used as a reference, and all video sequences were standardized to 5300 frames. Consequently, each video was represented as a three-dimensional array with dimensions (222, 5300, 468), comprising 222 lifting trials, 5300 temporal points per trial, and trajectories of 468 facial landmarks per temporal point. Zero-padding was implemented for videos shorter than 5300 frames to maintain consistent array dimensions across all samples.

### 2.5. Bio-Signals Dataset

**Figure 2:**
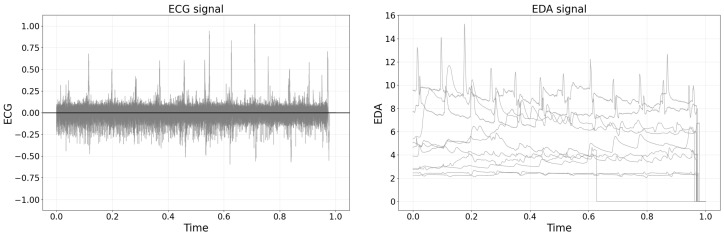
(**Left**) Raw ECG signal demonstrating rhythmic heartbeats during lifting tasks. (**Right**) Raw EDA signal from the neck region, which shows minimal artifacts despite the susceptibility to motion.

Raw bio-signal data were obtained using the OpenSignals platform and subsequently saved as text files. As shown in Figure 2, the raw EDA and ECG signals were plotted by randomly sampling 10 recordings from the total 222 datasets. For the analysis, raw data were intentionally selected over pre-processed features, a decision motivated by prior research [5] highlighting potential uncertainties and errors introduced by manual feature engineering. Moreover, the extraction of numerous features from a single bio-signal can result in high inter-variable correlations, complicating interpretability and potentially diminishing classification performance. Therefore, utilizing raw bio-signal data was deemed more advantageous for ensuring analytical robustness.

The raw text files initially comprised semi-structured data, organized yet unsuitable for direct analysis. Consequently, Python scripting was employed to convert the data into structured tabular formats. Each dataset consisted of two columns corresponding to ECG and EDA signals, with row numbers determined by task duration in seconds multiplied by the sampling frequency of 300 Hz.

To identify potential outliers, Tukey’s method, utilizing the interquartile range (IQR), was applied [6]. Data points falling beyond 1.5 times the IQR above the third quartile or below the first quartile were considered outliers. However, no outliers were detected in either the ECG or EDA datasets.

It is possible that recording EDA signals from the neck region could be susceptible to motion artifacts, as indicated by prior studies. In addition to using Tukey’s method based on the IQR to identify potential statistical outliers, the authors visually inspected all bio-signal recordings for characteristic signs of motion artifacts, such as sudden spikes, abrupt discontinuities, or unusually rapid signal fluctuations inconsistent with physiological norms. Trials exhibiting clear evidence of substantial motion-related distortion or data corruption were carefully reviewed and excluded from subsequent analysis. Although more sophisticated methods such as accelerometer-based artifact detection or adaptive filtering methods were not implemented in this study, future work should consider integrating these additional strategies.

The left plot of Figure 2 shows a representative raw ECG signal recorded during a manual lifting task, demonstrating distinct heartbeat peaks amid baseline fluctuations typically observed in biosignal measurements. The right plot of Figure 2 presents representative raw EDA signals from multiple participants, illustrating both the individual variability in baseline skin conductance and transient changes associated with physiological responses during lifting tasks.

### 2.6. Bio-Signal Data Synchronization and Dataset

Considering that bio-signals were recorded at a frequency of 300 Hz while video recordings were captured at 30 frames per second, synchronization of the datasets was required. Bio-signals were therefore downsampled by averaging every ten consecutive data points into a single value, as described by Equation (2):

(2) $$x_{i}=\frac{1}{10}\sum_{j=1}^{10}y_{10\times (i-1)+j}$$

For example, $x_{1}$ denotes the mean of the initial ten bio-signal measurements ($y_{1},y_{2},\ldots ,y_{10}$), while $x_{2}$ corresponds to the subsequent set of values ($y_{11},y_{12},\ldots ,y_{20}$). This downsampling procedure aligned bio-signal data with the video frame rate of 30 data points per second, facilitating synchronization between the two datasets.

In this study, a moving average filter in (2) is employed to downsample bio-signal data from 300 Hz to 30 Hz. This approach was selected primarily for synchronization with video data captured at 30 frames per second and to reduce the high-frequency noise inherent in physiological signals. The moving average method operates by averaging consecutive samples within a sliding window, effectively attenuating rapid fluctuations and enhancing the signal’s underlying physiological trends. Due to its computational simplicity, intuitive implementation, and proven effectiveness in reducing measurement artifacts, moving average filtering is widely adopted in biosignal preprocessing applications. The authors also considered alternative methods such as decimation and interpolation, but these methods typically do not inherently mitigate noise and might inadvertently amplify artifacts or introduce artificial fluctuations. Therefore, the moving average filter was the most appropriate for preserving meaningful low-frequency physiological content while achieving synchronization and improving signal quality.

Following synchronization, the bio-signal dataset was structured into a three-dimensional tensor with dimensions (222, 5300, 2), where the first dimension represents 222 lifting trials, the second dimension comprises 5300 temporal points per trial, and the third dimension corresponds to the two physiological channels (ECG and EDA).

### 2.7. Dataset Configurations for Evaluating Modalities and Feature Selection

To assess the contribution of each modality and examine the impact of feature selection, five distinct dataset configurations were evaluated:

Dataset 1: Facial only
includes trajectories of 468 facial landmarks (5300×468), excluding bio-signals.
Dataset 2: Bio-signals only
comprises two synchronized bio-signals (ECG and EDA) without facial landmark features.
Dataset 3: All features
Integrates a full fusion of both facial landmarks and bio-signals (5300×470).
Dataset 4: Selected facial
utilizes 12 facial trajectories selected via the classif.gsam.vs algorithm (refer to Section 2.7), without bio-signals.
Dataset 5: Selected facial + bio-signals
combines the 12 selected facial trajectories from Dataset 4 with the two bio-signals, forming a tensor of dimensions (5300×14).

These configurations enable a comparative analysis among: (i) a facial-landmarks-only model (Dataset 1), (ii) a bio-signals-only model (Dataset 2), (iii) a complete fusion model (Dataset 3), and (iv) two dimensionally reduced variants based on selected facial landmarks (Datasets 4 and 5). The number of features (*p*) ranges accordingly from p=2 (bio-signals only) to p=12 (selected facial landmarks), p=14 (selected facial landmarks combined with bio-signals), p=468 (all facial landmarks), and p=470 in the fully integrated dataset. Consequently, each lifting trial is represented by *p* synchronized functional trajectories, denoted by $X_{1}\left(t\right),\ldots ,X_{p}\left(t\right)$, for t∈[0,1].

#### Facial Variable Selection

For Dataset 4 (selected facial) and Dataset 5 (selected facial combined with bio-signals), the dimensionality reduction of the 468 facial landmark trajectories was performed using the classif.gsam.vs available in the fda.usc R package [7,8]. This method implements a functional generalized additive model (FGAM) employing a forward–backward (stepwise) selection strategy (detailed in Section 3.3), iteratively adding the trajectory whose basis coefficients yield the greatest reduction in multinomial deviance and subsequently removing any non-significant terms. The procedure incorporates five-fold internal cross-validation to mitigate overfitting. When applied to the training data of the initial data split, the algorithm consistently selected the same set of 12 landmark trajectories. Consequently, these 12 landmark indices were predetermined and uniformly applied across all 100 simulation runs, ensuring comparability and consistency throughout the experimental evaluations.

## 3. Method

FDA is a statistical framework specifically developed for analyzing data observed as continuous functions over domains such as time or space [9]. Unlike conventional analytical methods that regard discrete observations independently, FDA explicitly models the underlying continuous function from which these observations originate. This approach leverages the inherent structure and smoothness of functional data and predictive accuracy. In FDA, a typical representation of an observed function X(t) employs a basis function expansion of the form

(3) $$\begin{array}{c} X\left(t\right)\approx \sum_{k=1}^{K}a_{k}\varphi_{k}\left(t\right) \end{array}$$

where ${\left\{\varphi_{k}\left(t\right)\right\}}_{k=1}^{K}$ denotes predefined basis functions (such as B-splines or Fourier bases) and $a_{k}$ values are coefficients indicating the contribution of each basis to the function [10]. Utilizing this basis function representation reduces dimensionality, mitigates noise, and maintains smoothness inherent in temporal or spatial data.

In the current research, FDA is employed to represent facial expression data and physiological bio-signals (ECG and EDA), capturing their continuous variations throughout lifting tasks. Instead of directly using high-dimensional, discretized data vectors, this study exploits FDA-based basis function expansions to derive meaningful, lower-dimensional features that effectively encapsulate the essential data characteristics.

To overcome the limitations of traditional FDA methods that rely on fixed, pre-selected basis functions, this study integrates an adaptive neural network architecture incorporating learnable basis functions through a specialized basis layer. This adaptive FDA methodology facilitates task-specific representation learning, enabling the model to highlight and exploit patterns most pertinent to ergonomic risk classification in lifting tasks [11].

### 3.1. AdaFNN

To effectively model functional inputs, this study utilizes the Adaptive Functional Neural Network (AdaFNN), a specialized neural architecture developed explicitly for FDA [11]. Traditional FDA methods generally employ fixed basis expansions, such as B-splines or Fourier series, to reduce data dimensionality. However, such predefined bases do not consider the specific prediction task and thus may not optimally capture the features most relevant to the predictive outcome.

AdaFNN addresses this constraint by introducing an adaptive basis layer (BL), wherein each basis function is represented by an individual micro-neural network. Rather than preselecting basis functions, the AdaFNN adaptively learns optimal basis functions during training through backpropagation. Each micro-network maps time points t∈[0,1] to scalar basis values $\beta_{i}\left(t\right)$. The inner product between a basis function $\beta_{i}\left(t\right)$ and an input function X(t) is computed as

(4) $$c_{i}=\langle \beta_{i},X\rangle =\int \beta_{i}\left(t\right)\cdot X\left(t\right),dt.$$

These inner products $\left\{c_{i}\right\}$ form a compact, low-dimensional vector representation, which is subsequently processed through fully connected layers for predictive modeling.

To enhance computational efficiency, AdaFNN incorporates two regularization terms: an orthogonality penalty, ensuring that the learned basis functions capture distinct, non-redundant features, and a sparsity penalty.

AdaFNN employs an end-to-end training strategy, enabling the simultaneous learning of functional representations and predictive relationships. This methodology provides superior classification performance and more concise data representations compared to traditional FDA approaches.

### 3.2. Multi-AdaFNN

**Figure 3:**
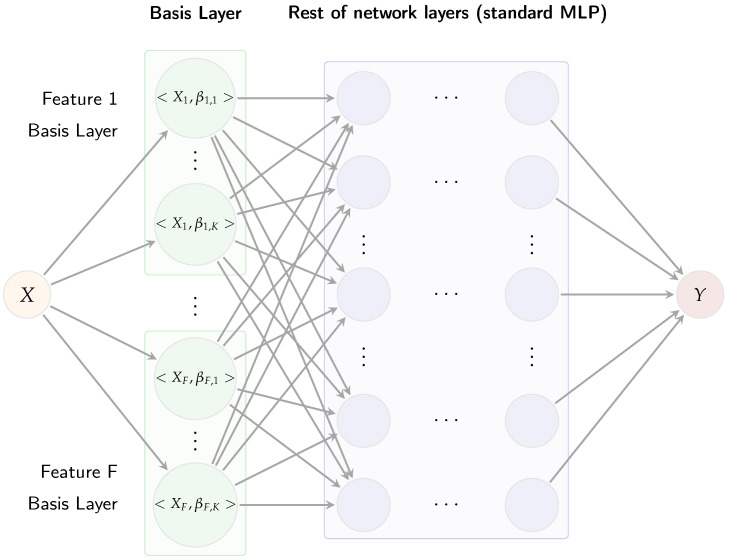
Illustration of the Multi-AdaFNN neural network architecture.

The original AdaFNN architecture was developed primarily for univariate functional predictors [11]. In contrast, the dataset comprises multivariate functional observations structured as a tensor of dimensions (n,J,F), representing *n* trials, J=5300 temporal points, and *F* features. To address this complexity, this study extends AdaFNN into a multi-branch architecture, designated as Multi-AdaFNN, wherein each feature (or channel) receives a distinct adaptive BL. Figure 3 illustrates the Multi-AdaFNN, organized sequentially into three main components: an adaptive basis layer, coefficient fusion, and a multi-layer perceptron (MLP) classifier.

The adaptive basis layer assigns *K* learnable micro-networks to each input feature trajectory, generating inner-product coefficients. Each BL is implemented through multiple compact neural networks, termed micro-networks [11]. Specifically, for each input feature f∈{1,…,F}, a dedicated basis layer comprising *K* micro-networks is constructed. Each micro-network receives a time value *t* as input and produces a scalar output $\beta_{f,k}\left(t\right)$. Evaluating these micro-networks across all sample points yields *K* basis functions $\left\{\beta_{f,1}\left(t\right),\ldots ,\beta_{f,K}\left(t\right)\right\}$ for each feature. Each micro-network is a two-layer perceptron with hyperbolic tangent (tanh) activations to ensure smooth and bounded outputs, followed by a linear output layer allowing basis values $\beta_{f,k}\left(t\right)$ to assume both positive and negative values. For an input curve $X_{f}\left(t\right)$, the corresponding micro-network coefficient $c_{f,k}$ is calculated as

(5) $$\begin{array}{c} c_{f,k}=\langle \beta_{f,k},X_{f}\rangle =\int_{0}^{1}\beta_{f,k}\left(t\right)X_{f}\left(t\right)\;dt, \end{array}$$

approximated numerically via the trapezoidal rule. Concatenating all coefficients from the *K* bases for each feature results in a coefficient vector $c\in R^{K\times F}$. In this paper, the same number *K* of basis functions is used for each feature to ensure balanced representation, and AdaFNN’s regularization terms are included: an $\ell_{1}$ sparsity penalty promoting localized basis functions and an $\ell_{2}$ cosine similarity penalty enforcing orthogonality among learned bases.

The resulting coefficient vector *c* subsequently feeds into a two-hidden-layer MLP classifier. Each hidden layer employs rectified linear units (ReLU) activations followed by layer normalization, enhancing training stability when coefficient scales vary across modalities [11]. The final layer of the MLP contains three output neurons corresponding to the three risk categories, with softmax activation generating predicted class probabilities $\hat{y}=\mathrm{softmax}\left(W_{\mathrm{out}}h+b_{\mathrm{out}}\right)$, where *h* represents the final hidden layer outputs.

In summary, Multi-AdaFNN systematically converts multivariate time-series data $X\left(t\right)=[X_{1}\left(t\right),\ldots ,X_{F}\left(t\right)]$ into a concise feature representation *c* via adaptive functional basis layers, subsequently classifying this representation through an MLP classifier. The joint training of the adaptive basis layer and MLP classifier ensures gradient propagation throughout the entire architecture, refining the learned basis functions $\beta_{f,k}\left(t\right)$ to emphasize predictive patterns critical for risk classification [11]. Unlike traditional fixed-basis FDA methods, this adaptive methodology directly optimizes the functional bases specifically for accurate risk discrimination.

A detailed step-by-step description of the forward pass for a single trial is presented in Algorithm 1.
**Algorithm 1:** Forward Pass of Multi-AdaFNN.
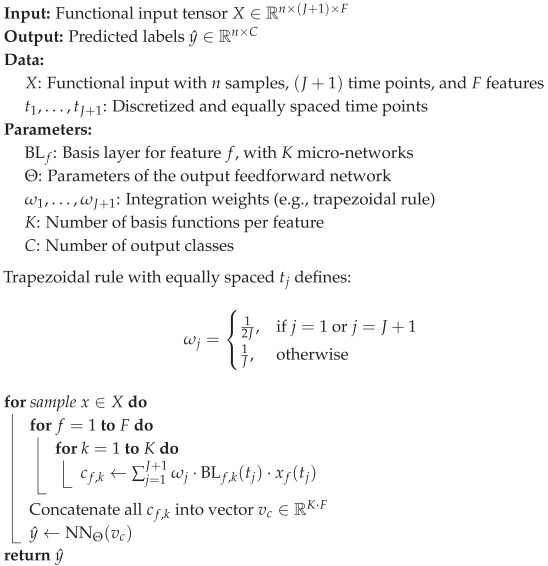


### 3.3. GSAM

Generalized additive models (GAMs) extend classical generalized linear models by replacing linear predictors with smooth functions $f_{i}\left(\cdot \right)$ of corresponding covariates [12]. When dealing with functional predictors represented by trajectories X(t), this concept leads to functional generalized additive models (FGAMs), which model the effect of each trajectory via smooth functions applied to their basis coefficients [13]. Using a canonical link function g(·), scalar covariates $Z_{i}$, and basis coefficients ${\left\{\xi_{j}^{\left(k\right)}\right\}}_{j=1}^{K_{k}}$ of the *k*-th functional predictor, an FGAM can be expressed as

(6) $$\begin{array}{c} g\left\{E\left[Y\right]\right\}=\alpha +\sum_{i}f_{i}\left(Z_{i}\right)+\sum_{k=1}^{q}\sum_{j=1}^{K_{k}}f_{j}^{\left(k\right)}\left(\xi_{j}^{\left(k\right)}\right), \end{array}$$

where each smooth function $f_{i}$ or $f_{j}^{\left(k\right)}$ is estimated using penalized regression splines. Ref. [13] proposed an enhanced version of FGAM incorporating automatic variable selection. The GSAM implementation is available in the fda.usc R package. Here, classif.gsam.vs conducts a forward–backward selection procedure on candidate functional predictors (curves or landmarks) to minimize out-of-sample deviance derived from multinomial logistic loss. At each iteration, the algorithm computes distance correlation (or an analogous score-test statistic) between the remaining candidate trajectories and residuals, progressively selecting the most informative trajectories. It subsequently re-optimizes smoothness parameters and prunes non-significant terms until no further model improvement is observed [7,8]. Each time a trajectory is incorporated, a nested generalized likelihood-ratio test determines whether its effect should remain linear to reduce complexity or be modeled using nonlinear spline terms.

Application of classif.gsam.vs to the 468 facial-landmark trajectories from the training fold of the initial data split consistently identified the same subset of 12 landmarks. These landmark indices were subsequently fixed and utilized uniformly across all simulations, forming the reduced set of facial features employed in Datasets 4 and 5.

### 3.4. Training Procedure

The Multi-AdaFNN model was trained using a weighted cross-entropy loss function to effectively manage class imbalance. Specifically, let $w_{0},w_{1},w_{2}$ represent the class-specific weights for the three risk levels labeled as 0, 1, and 2, respectively. Initially, weights were determined inversely proportional to the class frequencies in the training set, defined as $w_{c}=\frac{1}{N_{c}}$, where $N_{c}$ denotes the number of training samples belonging to class *c*. Subsequently, these weights were normalized so that $\sum_{c}w_{c}=3$. Consequently, the penalty for misclassifying instances from less frequent classes (e.g., high-risk class 2) became more significant relative to the common class (low-risk class 0). For each training sample with true class label *c*, the loss *L* was computed as

(7) $$\begin{array}{c} L=-w_{c}\log{\hat{p}}_{c}, \end{array}$$

where ${\hat{p}}_{c}$ is the predicted probability of the true class. This weighting scheme mitigates the tendency of the network to disproportionately favor the majority class. The model was implemented using the PyTorch framework and optimized through the Adam optimization algorithm, configured with a learning rate of 0.0001, $\beta_{1}=0.9$, and $\beta_{2}=0.999$ [14]. Each model run was trained for a maximum of 500 epochs, incorporating an early stopping criterion wherein training was terminated if no improvement in validation loss was observed over 20 consecutive epochs, thereby preventing overfitting. To further enhance generalization performance and manage model complexity, regularization methods were employed. Dropout regularization with a dropout rate of 20% was applied within the classifier MLP layers to reduce overfitting [15]. The adaptive basis layers also included specialized regularization penalties such as an $\ell_{1}$ sparsity penalty to promote localized basis functions, and an $\ell_{2}$ orthogonality penalty to minimize redundancy among learned bases. Careful architectural decisions were also made to stabilize training across the complex multi-branch architecture. Layer normalization was applied within adaptive basis layers to achieve uniform scaling and stable gradient propagation [16]. Activation functions were chosen methodically, with tanh activations employed in micro-networks to generate smooth and continuous basis functions and ReLU used in classifier layers for effective non-linear feature extraction [17]. Hyper-parameter tuning was also conducted using a stratified 3-fold cross-validation approach, systematically evaluating various parameters such as the number of basis functions, micro-network widths, MLP layer dimensions, and regularization strengths. This optimization procedure ensured that the final model configuration achieved an optimal balance between complexity and predictive performance, demonstrating strong generalization capabilities, as evidenced by robust outcomes across repeated simulations.

### 3.5. Hyper-Parameter Tuning

For hyper-parameter tuning, the dataset was partitioned into a stratified training set comprising 70% of the data and a hold-out test set containing the remaining 30%. Within the training subset, stratified 3-fold cross-validation was conducted to determine the optimal hyper-parameter configuration that maximized the average weighted F1-score across folds. These optimal settings were then consistently applied to all subsequent simulation runs. The hyper-parameter tuning process was conducted in two sequential stages.

Initially, the impact of activation functions on both optimization efficiency and the shape of the learned basis functions was explored. Four activation function pairs {(tanh,ReLU),(tanh,SELU),(ReLU,ReLU),(SELU,SELU)} were systematically evaluated, where the first function in each pair corresponds to the activation of the micro-network and the second corresponds to the MLP classifier activation. The combination of tanh for the basis layer and ReLU for the MLP classifier achieved the highest mean weighted F1-score and exhibited the most stable training dynamics. This combination was thus adopted for all subsequent analyses.

With activation functions fixed, the second stage involved tuning capacity and regularization hyper-parameters. Specifically, three primary capacity-related hyper-parameters were optimized: (i) the number of basis functions per feature *K*, (ii) the width of each micro-network, and (iii) the depth and width of the classifier MLP. Each parameter was evaluated using a coarse-to-fine grid search across three configurations (small, medium, and large). Performance metrics consistently improved up to the medium configuration and plateaued thereafter; hence, the smallest configuration within this plateau was selected to promote computational efficiency. Regularization hyper-parameters $\lambda_{1}$ (controlling sparsity) and $\lambda_{2}$ (controlling orthogonality) were jointly tuned via a three-level grid search to ensure basis functions remained sparse and distinct without adversely affecting the AUC. In addition, the sampling parameters $k_{R_{1}}$ and $k_{R_{2}}$, which specify the number of basis functions subsampled when computing regularization penalties $R_{1}$ and $R_{2}$, respectively, were also optimized. Drop-out regularization rates of 0, 0.10, and 0.20 were assessed. Layer normalization was consistently retained throughout all experiments, as its removal occasionally resulted in training divergence. Residual (skip) connections around each MLP hidden layer were uniformly employed, as preliminary experiments demonstrated enhanced convergence rates without affecting optimal hyper-parameter selection.

The described two-stage grid search was conducted independently for each of the five dataset configurations. Consequently, the optimal hyper-parameter configurations reported in Table 3 are tailored specifically to their respective datasets.

### 3.6. Evaluation of Model Generalization Through Repeated Stratified Simulations

To achieve robust estimation of the model’s generalization capability, a total of 100 independent simulation runs were conducted. For each simulation, the entire dataset was stratified into a training subset (70%) and a hold-out testing subset (30%). Hyper-parameter tuning was performed exclusively on the training subset following the methodology described in Section 3.5, and the resulting optimal hyper-parameters were then fixed. With these selected hyper-parameters, the Multi-AdaFNN model was subsequently trained using the entire training subset and evaluated exactly once on the corresponding testing subset. Performance metrics obtained from each of the 100 runs were aggregated to compute the mean and standard deviation, thereby avoiding data leakage from the testing subset during hyper-parameter optimization and accurately capturing performance variability due to different data splits.

### 3.7. Evaluation Metrics

Classification performance was assessed using accuracy, weighted F1-score, and AUC. Accuracy measures the overall proportion of correctly classified instances; however, it can be misleading in scenarios involving class imbalance. Consequently, the weighted F1-score was computed using the average=’weighted’ option provided by scikit-learn [18], where each class-specific F1-score is proportionally weighted according to class prevalence, mitigating the dominance of majority classes. Additionally, AUC was calculated for each class in a one-versus-rest manner, with an average AUC subsequently reported. AUC, being threshold-independent, is particularly effective in evaluating the ranking quality of predicted probabilities, providing insight into the model’s ability to consistently assign higher risk probabilities to genuinely higher-risk trials under conditions of imbalance [19]. While class-specific precision and recall were occasionally examined, primary comparisons and inferences were based on the AUC metric due to its robustness in reflecting model performance in imbalanced classification contexts. All performance metrics were computed on the test sets of each independent simulation run and then averaged to yield final reported values.

### 3.8. Baseline Model

In addition to the proposed Multi-AdaFNN, an LSTM was implemented as a baseline. The LSTM used the same architecture search range, tuning procedure (3-fold CV), and optimization settings (Adam, learning rate, early stopping) as Multi-AdaFNN. The performance of the model was aggregated over 100 independent runs.

## 4. Results

### 4.1. Single-Modality vs. Multi-Modality

Table 4 presents a comprehensive summary of the classification performance metrics (mean ± SD) for the Multi-AdaFNN model across 100 independent simulation runs, evaluated on the five different dataset configurations. A potential source of bias in risk-level prediction arises when data are partitioned on a per-sample rather than a per-subject basis. In this study, each subject contributes multiple recordings, so sample-level partitioning of the dataset would allow information from the same individual to appear in both training and in evaluation sets, leading to data leakage and overly optimistic performance estimates [20]. To investigate and address this concern, this study ran 100 independent random-split simulations—each time dividing the entire dataset at the sample level into training and testing subsets (and similarly for each fold of three-fold cross-validation)—and recorded the accuracy, weighted F1-s, core and AUC for every run. Table 4 shows low variance and consistently strong performance, demonstrating that the model’s accuracy is not an artifact of data leakage but instead reflects genuine generalizability, even under sample-level partitioning. In general, the multi-modal fusion approach, represented by Dataset 3, which integrates all available facial landmark trajectories with bio-signal data (ECG and EDA), consistently achieves superior performance across all evaluated metrics. In particular, Dataset 3 yielded the highest mean accuracy (0.6370 ± 0.0589), the highest weighted F1-score (0.7546 ± 0.0571), and the highest AUC (0.6320 ± 0.0599), significantly outperforming single-modality configurations. These findings underscore the clear benefit and added value provided by fusing multiple sources of temporal data for ergonomic risk classification tasks.

In contrast, relying exclusively on facial landmarks (Dataset 1) resulted in lower performance (accuracy: 0.6164 ± 0.0548; weighted F1-score: 0.7299 ± 0.0547; AUC: 0.6114 ± 0.0552). Similarly, utilizing only bio-signals (Dataset 2) led to even more substantial performance degradation (accuracy: 0.4352 ± 0.0910; weighted F1-score: 0.5093 ± 0.0516; AUC: 0.3179 ± 0.0834). The considerable performance gap observed between these single-modality approaches and the fully integrated multi-modal dataset highlights the complementary nature of facial expressions and physiological signals, indicating that each modality captures distinct yet complementary aspects of ergonomic risk.

Moreover, the fully fused model (Dataset 3) demonstrated greater consistency, as reflected by the lower standard deviations across performance metrics, suggesting enhanced stability and reliability compared to single-modality configurations. This robustness is crucial for practical applications in real-world ergonomic monitoring scenarios, where consistent performance is essential for reliable decision-making.

### 4.2. Effect of Dimensionality Reduction

Further analysis of dimensionally reduced datasets also provides meaningful insights. Dataset 4, employing a subset of 12 selected facial landmarks obtained through the GSAM variable selection method, exhibited a performance (accuracy: 0.4355 ± 0.0543; weighted F1-score: 0.6363 ± 0.0663; AUC: 0.4122 ± 0.0779) slightly below that of the complete facial dataset, suggesting that while the selected features capture significant aspects of facial expression variations relevant to risk prediction, additional landmarks may offer incremental predictive value. Interestingly, Dataset 5, which combined these selected facial landmarks with bio-signals, demonstrated improved performance relative to Dataset 4 alone (accuracy: 0.4485 ± 0.0636; weighted F1-score: 0.6480 ± 0.0622; AUC: 0.4421 ± 0.0768), though still falling short of the comprehensive multi-modal fusion approach (Dataset 3). This incremental improvement further validates the advantage of multi-modal integration, emphasizing that even selected subsets of features, when supplemented by complementary bio-signals, can notably enhance model performance.

These empirical results collectively highlight that comprehensive multi-modal data integration—particularly the fusion of full facial landmark trajectories with bio-signals—yields the most effective ergonomic risk classification outcomes, evidenced by superior overall accuracy, balanced classification performance, and reduced variability. Consequently, Multi-AdaFNN emerges as a robust, reliable, and practically relevant framework for effectively combining and leveraging multi-modal temporal data sources in ergonomic risk assessment tasks.

### 4.3. ROC Curve Analysis

**Figure 4:**
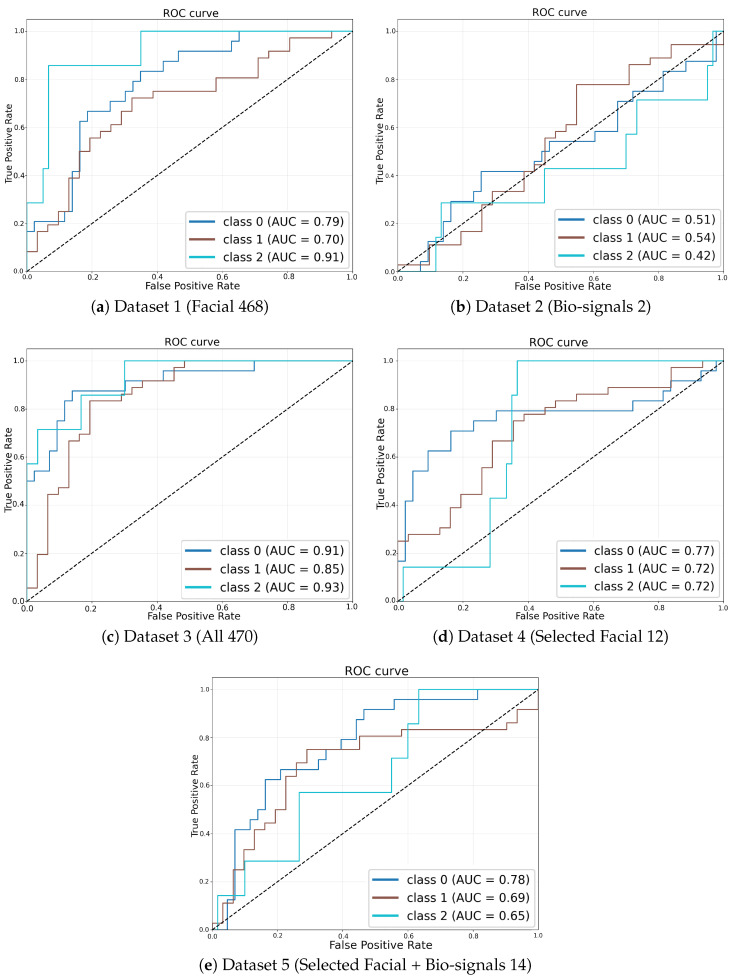
ROC curves corresponding to the simulation runs with the highest weighted F1-score for each dataset configuration.

Figure 4 illustrates ROC curves derived from the simulation runs that achieved the highest weighted F1-score within each dataset configuration, providing a clear visual assessment of classifier performance. The ROC curve corresponding to the fully integrated multi-modal dataset (Dataset 3, Figure 4c) encloses the largest area, underscoring its superior discriminative capability compared to the other configurations. In contrast, ROC curves associated with single-modality models, namely the facial-landmark-only model (Dataset 1, Figure 4a) and the bio-signals-only model (Dataset 2, Figure 4b), exhibit notably lower true positive rates at corresponding false positive rates, situated significantly closer to the diagonal line indicative of random-guessing performance.

Moreover, the multi-modal configuration utilizing selected facial landmark trajectories combined with bio-signals (Dataset 5, Figure 4e) also demonstrates improved discriminative performance relative to the single-modality configurations, though it remains slightly inferior to the full fusion approach (Dataset 3). This observation further validates the incremental value provided by combining reduced-dimensionality facial features with bio-signal data.

All dataset configurations exceed chance-level classification performance, as evidenced by their ROC curves consistently surpassing the diagonal no-discrimination baseline. However, the superiority and effectiveness of multi-modal data fusion over single-modality inputs are particularly pronounced in these visual comparisons. These ROC-derived insights closely align with the numerical AUC results presented in Table 4, wherein Dataset 3 achieves the highest mean AUC value of approximately 0.63, followed by Dataset 5. Both multi-modal fusion configurations substantially outperform the single-modality cases.

The ROC analysis compellingly demonstrates that the integration of facial landmarks with bio-signals in temporal sequence data markedly enhances the accuracy and robustness of ergonomic risk classification, reinforcing the quantitative findings depicted through aggregate performance metrics.

### 4.4. Quantitative Comparison and Statistical Testing

All statistical comparisons were conducted at a significance level of α=0.05. First, the Shapiro–Wilk test was applied to the 100 accuracy scores of each dataset to assess normality. Datasets 1, 3, 4, and 5 satisfied the normality assumption. Because each simulation run produces paired observations and the differences are approximately normal, paired *t*-tests were then used to compare their mean accuracies. For all comparisons among Datasets 1, 3, 4, and 5 (1 vs. 3, 1 vs. 4, 1 vs. 5, 3 vs. 4, 3 vs. 5, and 4 vs. 5), the *t*-statistic exceeded the critical value with p<0.05, leading to rejection of the null hypothesis of equal means.

Dataset 2 violated the normality assumption and was therefore analyzed using the Wilcoxon signed-rank test, a nonparametric alternative that accommodates paired samples without assuming normality. Dataset 2 differed significantly from Datasets 1 and 3 ($W_{1,2}\;=\;18,p=1.443\times 10^{-17}$; $W_{2,3}=26,p=1.250\times 10^{-17}$) but showed no significant difference when compared to Datasets 4 or 5 ($W_{2,4}=2471.5,\;p=0.9903$; $W_{2,5}=2037.0,\;p=0.2876$). These findings indicate that the fully fused multi-modal configurations (Datasets 1, 3, 4, 5) exhibit statistically significant differences in mean accuracy, whereas the bio-signals-only configuration (Dataset 2) only differs significantly from the facial-landmarks-only and full-fusion setups and not from the reduced-feature variants.

### 4.5. Comparison with an LSTM Baseline

Table 5 presents the LSTM model’s classification performance—accuracy, weighted F1-score, and AUC—for each dataset configuration, with results reported as mean ± standard deviation over 100 simulation runs. Performance was highly degenerate: on Datasets 2 and 5, the standard deviation of all metrics was zero, indicating that the model defaulted to predicting the majority class rather than learning meaningful patterns. Across all five datasets, the LSTM yielded accuracies from 0.3582 (Dataset 5) to 0.5373 (Dataset 2), weighted F1-scores clustered tightly around 0.50, and AUC values between 0.1889 and 0.3756. By contrast, the Multi-AdaFNN (Table 4) achieved substantially higher accuracy, weighted F1-score, and AUC on every dataset, demonstrating its superior ability to capture the underlying temporal and multi-modal structure of the inputs.

### 4.6. Execution Time

Execution times were measured over 50 independent simulation runs per dataset (Table 6). Dataset 1 required on average 19 min and 13 s (±3 min and 32 s) for the Multi-AdaFNN model, and 1 min and 26 s (±19 s) for the LSTM. For Dataset 2, both models completed in under 10 s (Multi-AdaFNN: 7 s ± 4 s; LSTM: 7 s ± 2 s). For Dataset 3, Multi-AdaFNN ran for 20 min and 3 s (±3 min and 58 s), compared to 1 min and 1 s (±16 s) for the LSTM. In contrast, Dataset 4 yielded 1 min and 21 s (±27 s) for Multi-AdaFNN and 2 min and 10 s (±40 s) for the LSTM, while Dataset 5 showed execution times of 1 min and 29 s (±36 s) for Multi-AdaFNN and 20 s (±7 s) for LSTM.

## 5. Discussion

### 5.1. Benefits of Multi-Modal Fusion

The experimental results highlight the efficacy and significance of employing multi-modal data fusion for ergonomic risk classification. In particular, the superior performance demonstrated by the fully integrated multi-modal configurations (Datasets 3 and 5) underscores the complementary nature of combining visual and physiological information. Practically, facial landmark trajectories capture external indicators of physical strain or discomfort, such as subtle changes in facial expressions or head and neck posture variations. Simultaneously, bio-signals, including ECG and EDA, offer quantifiable internal physiological responses reflective of stress and workload. The Multi-AdaFNN framework effectively integrates these distinct data sources, enabling the model to detect complex risk-related patterns that single-modality approaches might fail to capture. For instance, during high-risk lifts, subtle facial movements combined with pronounced physiological responses like heart rate acceleration and elevated electrodermal activity provide critical cues. A facial-only approach might overlook scenarios involving minimal visible expression changes, whereas a bio-signal-only model could misinterpret cases lacking prominent physiological responses. The superior weighted F1-score obtained with multi-modal fusion further emphasizes the model’s enhanced capability to accurately distinguish between all risk categories, including the particularly challenging intermediate-risk cases. Hence, multi-modal integration not only boosts overall classification accuracy but also delivers a more comprehensive and reliable assessment of ergonomic risks during lifting tasks.

### 5.2. Performance Trade-Offs and Challenges

Despite the clear advantages demonstrated by Multi-AdaFNN, the overall magnitude of the performance metrics highlights inherent challenges associated with ergonomic risk classification tasks. The best-observed AUC was approximately 0.63, accompanied by similar moderate ranges of accuracy and weighted F1-scores. While modest relative to typical binary classification results, this level of performance remains practically meaningful in the context of a three-class ergonomic risk prediction scenario, where distinguishing nuanced differences between low, medium, and high risk levels is inherently complex. Medium-risk tasks may share overlapping characteristics with low- or high-risk scenarios, and substantial inter-subject variability in both facial expressions and physiological responses introduces further complexity and ambiguity. Thus, an AUC score in the low-to-mid 0.60 s substantially surpasses random chance performance (AUC = 0.5), suggesting the model effectively captures significant, albeit subtle, distinctions within noisy and highly variable data. Furthermore, the achieved weighted F1-scores, ranging from around 0.51 (bio-signals-only) to 0.75 (fully fused data), confirm the classifier’s consistent ability to differentiate correctly between risk categories. Though there remains scope for performance improvement—particularly within single-modality approaches—the established baseline performance provides a solid foundation for further model refinement, such as incorporating additional expressive features or expanding dataset sizes to better represent subtle risk-level variations.

### 5.3. Limitations and Future Work

Several limitations of this study should be acknowledged. First, the dataset employed for model training and evaluation was modest in scale, comprising a limited number of participants and trials. Although multiple stratified splits and averaging across simulations provided robustness, a larger dataset would enhance generalizability and the ability to capture more nuanced patterns distinguishing ergonomic risk levels. Secondly, the experimental conditions, though carefully controlled, may limit generalizability to real-world settings where numerous additional variables—such as varying load weights, diverse body mechanics, cumulative fatigue, and environmental constraints—significantly influence facial expressions and physiological responses. For instance, facial landmark tracking accuracy could diminish in uncontrolled lighting conditions or when facial visibility is obstructed by protective equipment. Similarly, bio-signal quality might degrade due to motion artifacts or sensor placement inconsistencies in industrial settings. To detect motion artifacts, future studies may consider incorporating simple rule-based signal quality assessment methods, such as the approach proposed by [21], which demonstrated strong agreement with expert evaluations in ambulatory EDA recordings. Additionally, the complexity of implementing the Multi-AdaFNN architecture practically, involving synchronized multi-sensor data collection and real-time analysis capabilities, poses potential challenges regarding hardware efficiency, computational demands, and user compliance with sensor wearability. Nevertheless, this research serves as a crucial proof of concept, demonstrating that a well-designed multi-modal approach can successfully identify meaningful indicators of ergonomic risk even with limited data. These insights encourage future research aimed at overcoming identified limitations, including expanding the dataset to encompass broader lifting scenarios and enhancing the robustness and practicality of the sensor integration and modeling processes for deployment in real-world occupational environments.

Although the proposed approach demonstrated promising performance in classifying ergonomic risks, this study acknowledges that the relatively small sample size (N=20) poses inherent limitations in the generalizability and robustness of the findings. Ergonomic risk assessments in practical settings typically involve considerable variability arising from multiple factors such as anthropometric differences, physiological variations, and diverse workplace conditions. The limited dataset in the current study may not fully capture this variability, potentially restricting the adaptability and accuracy of the model across different populations and work environments. To mitigate the impact of this limitation, several strategies have been implemented. Rigorous validation procedures involving repeated cross-validation have been employed to achieve robustness and reduce the risk of model overfitting [22]. Furthermore, regularization methods were incorporated during model training to further enhance the generalizability of the learned patterns. Nevertheless, to fully address the concerns regarding dataset limitations and enhance practical applicability, future studies should consider expanding the dataset by integrating data from multiple experimental sites and diverse participant groups. Methods such as domain adaptation, transfer learning, and data augmentation could be explored to improve the robustness of the model. Such extensions would facilitate capturing a broader range of ergonomic risks, enhancing the model’s adaptability to different groups of people and varying working environments.

There are limitations in the experimental protocol that merit further discussion. First, the features derived from facial landmarks and bio-signals may not exclusively represent direct measures of “lifting task risk levels” but could potentially capture general physiological responses, effort, or emotional reactions of subjects to the task conditions. In particular, facial landmarks could reflect broader emotional or stress-related states induced by the lifting scenarios, while physiological signals such as ECG and EDA may primarily reflect exertion levels or sympathetic nervous system activation rather than directly indexing ergonomic risk. Moreover, the categorization of ergonomic risk levels based on predefined task conditions (low, medium, or high) inherently involves assumptions about the physiological and emotional responses elicited by these conditions. Individual variations, including personal fitness levels, lifting techniques, and psychological factors, could significantly influence these responses, potentially introducing variability that is not strictly representative of task-defined risk levels. Therefore, interpretations drawn from the model should be considered cautiously, acknowledging that observed associations might reflect broader physiological or emotional responses rather than strictly ergonomic risk. Future studies should aim to include additional control measures or baseline comparisons to more clearly distinguish between general physiological effort or stress reactions and task-specific ergonomic risk indicators.

## 6. Conclusions

This study introduces and validates the Multi-AdaFNN model, effectively demonstrating its capability to integrate multi-modal functional data for ergonomic risk classification in manual lifting tasks. By jointly analyzing temporal trajectories of facial landmarks and bio-signals, the proposed model promotes predictive accuracy of risk levels. The adaptive basis layers within Multi-AdaFNN facilitate the identification and focus on the most informative patterns within each data modality, resulting in a fused feature representation that accurately captures lift dynamics associated with low, medium, and high ergonomic risk. The empirical results clearly indicate that multi-modal integration substantially outperforms single-modality approaches, confirming that visual cues from facial expressions and physiological indicators such as ECG and EDA provide complementary insights crucial for accurate risk classification.

The Multi-AdaFNN framework also presents promising practical implications for real-time ergonomic monitoring and proactive intervention in occupational settings. A real-time deployment scenario might involve the continuous monitoring of workers during manual lifting activities through unobtrusive wearable sensors and video-based facial tracking, automatically alerting individuals or supervisors when high-risk lifting patterns are detected. Such a proactive, data-driven approach could substantially mitigate musculoskeletal injury risks by promoting timely corrective actions, including rest breaks or ergonomic adjustments. For successful real-world implementation, future research directions include extensive validation on larger-scale, diverse datasets encompassing broader demographic populations, various lifting conditions, and environmental contexts to enhance model robustness and generalizability. In addition, technical advancements such as optimizing the model for efficient execution on edge computing devices or dedicated wearable hardware would significantly facilitate practical deployment, enabling effective on-site ergonomic risk monitoring independent of centralized computing resources. Overall, Multi-AdaFNN establishes a robust and practically applicable framework for multi-modal ergonomic risk prediction, holding substantial potential to enhance workplace safety through informed, real-time ergonomic interventions.

## Figures and Tables

**Table 1:** Experimental lifting conditions defined by combinations of VD and HD.

Condition	VD (cm)	HD (cm)
1	75	25
2	75	50
3	10	25
4	10	50

**Table 2:** Summary of experimental lifting tasks and associated risk levels based on the RWL defined by the RNLE.

Condition	VD (cm)	HD (cm)	RWL (kg)	Risk Categories
Condition 1	75	25	14.7	Low risk (Load weight < 14.7) Medium risk (14.7 < Load weight < 23)
Condition 2	75	50	7.3	Low risk (Load weight < 7.3) Medium risk (7.3 < Load weight < 14.7) High risk (14.7 < Load weight < 22)
Condition 3	10	25	12.0	Low risk (Load weight < 12) Medium risk (12 < Load weight < 23)
Condition 4	10	50	6.0	Low risk (Load weight < 6) Medium risk (6 < Load weight < 12) High risk (12 < Load weight < 18)

**Table 3:** Optimal hyper-parameter configurations for Multi-AdaFNN across the five dataset variants.

Dataset	Bases	Micro-Net	Classifier MLP	Drop-Out	$\lambda_{1}$	$\lambda_{2}$	$k_{R_{1}}$	$k_{R_{2}}$
Dataset 1	25	[64,32]	[384,192]	0.20	0.10	0.05	5	10
Dataset 2	15	[64,32]	[384,192]	0.00	0.30	0.10	4	3
Dataset 3	25	[64,32]	[384,192]	0.20	0.10	0.05	5	14
Dataset 4	110	[64,32]	[384,192]	0.20	0.10	0.05	5	20
Dataset 5	105	[64,32]	[384,192]	0.20	0.10	0.10	5	20

**Table 4:** Classification performance of Multi-AdaFNN across different dataset configurations: mean and standard deviation (SD) over 100 simulation runs; highest performance metrics highlighted in bold.

Dataset	Accuracy	Weighted F1-Score	AUC
Dataset 1	0.6164 ± 0.0548	0.7299 ± 0.0547	0.6114 ±0.0552
Dataset 2	0.4352 ± 0.0910	0.5093 ± 0.0516	0.3179 ± 0.0834
**Dataset 3**	**0.6370 ± 0.0589**	**0.7546 ± 0.0571**	**0.6320 ± 0.0599**
Dataset 4	0.4355 ± 0.0543	0.6363 ± 0.0663	0.4122 ± 0.0779
Dataset 5	0.4485 ± 0.0636	0.6480 ± 0.0622	0.4421 ± 0.0768

**Table 5:** Classification performance of LSTM across different dataset configurations: mean and standard deviation (SD) over 100 simulation runs.

Dataset	Accuracy	Weighted F1-Score	AUC
Dataset1	0.4424 ± 0.0898	0.5008 ± 0.0187	0.2767 ± 0.0936
Dataset2	0.5373 ± 0.0000	0.5003 ± 0.0096	0.3756 ± 0.0000
Dataset3	0.4621 ± 0.0888	0.4943 ± 0.0963	0.2972± 0.0926
Dataset4	0.4997 ± 0.0733	0.4999 ± 0.0515	0.3364 ± 0.0764
Dataset5	0.3582 ± 0.0000	0.5000 ± 0.0000	0.1889 ± 0.0000

**Table 6:** Average execution times of the Multi-AdaFNN and LSTM models across five datasets, computed over 50 independent simulation runs per dataset. Values are reported as mean ± standard deviation in HH:MM:SS format.

Dataset	Multi-AdaFNN	LSTM
Dataset 1	00:19:13 ± 00:03:32	00:01:26 ± 00:00:19
Dataset 2	00:00:07 ± 00:00:04	00:00:07 ± 00:00:02
Dataset 3	00:20:03 ± 00:03:58	00:01:01 ± 00:00:16
Dataset 4	00:01:21 ± 00:00:27	00:02:10 ± 00:00:40
Dataset 5	00:01:29 ± 00:00:36	00:00:20 ± 00:00:07

## Data Availability

The raw data supporting the conclusions of this article will be made available by the authors on request.
